# Examination of Ecological Systems Contexts Within a Latino-Based Community Sport Youth Development Initiative

**DOI:** 10.3389/fspor.2022.869589

**Published:** 2022-06-29

**Authors:** Mayra V. Robledo, Michael B. Edwards, Jason N. Bocarro, Andrew O. Behnke, Jonathan M. Casper

**Affiliations:** ^1^Department of Parks, Recreation & Tourism Management, North Carolina State University, Raleigh, NC, United States; ^2^School of Family & Consumer Sciences, Texas State University, San Marcos, TX, United States

**Keywords:** Latinx adolescents, community programs, sport-for-development (SFD), Juntos, qualitative evaluation, ecological systems theory

## Abstract

Youth Development Programs (YDPs) can serve as effective mechanisms to alleviate social and psychological adversities while enhancing and developing resilience among youth. Recently, more YDPs have incorporated sport within Sport for Development (SFD) models to achieve these goals. Due to the growing Latino population in the US and the wide achievement gap between Latinos and other demographic populations, there is a need to explore programs that may support individual development as well as long-term change with regard to social inequality. There is also a need to better understand the ecological contexts within SFD programs and how these contexts may support underserved youth. Specifically, using an ecological systems perspective, this study seeks to explore the implementation of a sport program by a YDP in order to examine the ecological processes that may support or inhibit the efficacy of sport programs working with underserved youth. Juntos is a YDP that primarily serves Latinx youth and families by assisting youth with graduating high school and pursuing higher education opportunities. Juntos incorporates two annual soccer tournaments (i.e., Kicking it with Juntos and Copa Unidos). A qualitative case study approach was implemented in two counties in North Carolina. Interviews were conducted with tournament participants, county coordinators and planning committee members. Findings found three key themes related to three ecological levels 1. At the Microsystem level, sport was implemented to engage youth and connect to non-sport program outcomes, but divergent perceptions of goals among stakeholders potentially inhibited intentional implementation. At the Mesosystem level, sport provided a mechanism to engage in collaborative relationships and encouraged parental participation. At the Macrosystem level, sport celebrated Latino culture and attempted to address social barriers facing Latinx youth, but some aspects of culture may have created barriers to access for girls. Findings suggested that while the programs emphasized mesosystem engagement, more integration across exosystem and macrosystem levels may be needed for sustainable outcomes.

## Introduction

In the United States (US), the academic gap between Latinx youth and other ethnic groups remains strikingly high. The US Hispanic student dropout rate of 7.7% is higher than that of Black students and nearly double that of White students (Irwin et al., 2021). Dropouts are more likely to have mental health issues, substance use, criminal behaviors, suicidal thoughts, and are prone to be arrested for larceny, assault, drug possession, or drug sales (Maynard et al., 2015). Many Latinos in the U.S. are in socially vulnerable positions. Structural factors such as anti-immigrant policies and poverty affect this population (Cabral and Cuevas, 2020). Latinos have been identified as a high-risk group for depression and anxiety connected to poverty, poor housing conditions, and rigid work demands (Magaña and Hovey, 2003). Latinx youth also experience significant barriers to academic achievement, including lower test scores and increased levels of school dropout (Harris and Kiyama, 2015). Many Latinx youth face significant academic challenges due to ethnic discrimination, limited resources, parent education, external pressures (peer and parental influences), and limited English language proficiency (Suárez-Orozco et al., 2010; Conchas, 2015). Youth Development Programs (YDPs) have been positioned as potential resources that can prevent and/or assist Latinx youth to navigate through these constraints, build competencies, and encourage resiliency (Borden et al., 2006).

Specifically, sport-based YDPs can be a context that promotes positive youth development (PYD) (Holt et al., 2020). The use of sport has been described by many researchers as having the potential to foster citizenship, responsibility, cooperation, leadership skills, social mobility, social cohesion, community integration, and positive peer relationships (Fraser-Thomas et al., 2005; Edwards, 2015; Jones et al., 2017). However, both Spaaij (2009) and Schulenkorf (2017) are critical of the simplistic notion that sport programs inherently lead to positive social, cultural, educational, economic, and health outcomes, a factor that was far too prevalent during the early years of Sport for Development (SFD) scholarship. In addition to programs needing to be designed to focus on PYD, developmental outcomes of sport participation are also contingent on contextual factors such as family, education, and the workforce (Spaaij, 2009). Thus, the positive relationship between sport and social, educational, cultural economic and health outcomes is not inherent and depends on the intentional implementation of the sport program that accounts for the participants' life context.

Bronfenbrenner's (1979) Ecological Systems Theory suggests that human development occurs through relationships among multiple processes of interaction between individuals and proximal contexts. Specifically related to PYD, scholars have noted that youths' pathway to becoming a fully functional adult is dependent not only on individual characteristics, but also through interactions with numerous contextual variables, including other individuals (e.g., peers, parents, and other adults), culture, and organizational and policy environments (Lerner, 2002). Due to the potential to bridge multiple contextual variables, youth sport may be a particularly salient setting for understanding how relevant ecological systems might interact in the process of PYD (Holt et al., 2008).

The combination of sport and YDPs has the potential to work well with youth populations because sport is one of the most popular activities among youth and youth sport programs, when intentionally designed to do so, have demonstrated developmental benefits (Fraser-Thomas et al., 2005; Pedersen and Thibault, 2014). Although there are significant benefits associated with youth sport, US Latinx youth are less likely than other racial groups to participate in sport (McGovern, 2021a). However, research has shown that soccer, because it offers a familiarized, comfortable, and cultural space, is a sport that could be used to engage Latinx youth (McGovern, 2021b). The incorporation of sport within YDPs, when it is implemented in innovative and culturally-relevant ways, has the potential to maximize the achievement of program goals (Cohen and Ballouli, 2018). Despite this promise, there is limited research on the linkage between YDPs (e.g., Boys and Girls Club, 4-H) and SFD within minority communities (Jones et al., 2017). Specifically, exploring how sport programs may facilitate processes related to ecological systems, unique to specific ethnic minority communities, may provide a better understanding of the contextual process that contribute to PYD. While scholars have studied positive youth development through sport among underserved youth, most of this research has focused either on refugee communities or programs located in the Global South, with fewer studies examining the context of racial and ethnic minority communities within the Global North (Whitley et al., 2015; Koopmans and Doidge, 2022). Additionally, more research is needed to understand how stakeholders across multiple levels of contextual influence engage with sport components of youth development (Whitley et al., 2015, 2018).

Due to the growing Latino population in the US and the wide achievement gap between Latinos and other demographic populations, there is a need to explore programs that may support individual development as well as long-term change with regard to social inequality. There is also a need to better understand the ecological contexts within SFD programs and how these contexts may support underserved youth. Specifically, using an ecological systems perspective, this study seeks to explore the implementation of a sport program by a YDP in order to examine the ecological processes that may support or inhibit the efficacy of sport programs working with underserved youth.

### Ecological Systems Theory in Youth Development

YDPs emerged following early Twentieth Century reforms that recognized a need in industrialized countries to protect and support children and adolescents, while also addressing neoliberal societal concerns of perceived youth delinquency and the breakdown of traditional family structures (Catalano et al., 2004). Often termed “After-school programs,” YDPs were primarily focused with preventing problem behaviors and providing constructive outlets for youth's leisure time. The focus of YDPs has evolved from being solely a preventative measure (e.g., focusing on fixing problems or deficits faced by youth), to shifting to developing the assets and strengths of young people, also known as Positive Youth Development (PYD) (Roth and Brooks-Gunn, 2003). From this perspective, YDPs attempt to build upon the existing strengths of youth and their environments to nurture assets (i.e., the values, relationships, and skills) needed for youth to develop into fully-functional adults (Catalano et al., 2004; Whitley et al., 2015). The most common factors behind successful YDPs is an approach to youth programming aimed at fostering positive development of youth into adulthood, program goals aligning with youth's needs, and healthy and stimulating environments (Barcelona and Quinn, 2016). One key criticism of youth development research and practice has been its emphasis on targeting and evaluating individual outcomes, often with a focus on short-term changes to behaviors and attitudes (Perkins et al., 2001). Particularly with YDPs working in communities of color and underserved youth, there is a need to recognize the complexity of the environments in which these youth engage with and how those environments may impact their development (Connell et al., 2001). Therefore, YDPs should be multilevel, providing opportunities to belong, model positive social norms, support efficacy and mattering, build skills, and provide a dynamic flow among various ecological systems (i.e., family, school, community) (Eccles and Gootman, 2002).

In their seminal report, Eccles and Gootman suggest that YDPs must connect across different levels of ecological systems in order to support overall youth development outcomes that apply beyond the short-term limits of program activities (Eccles and Gootman, 2002). Contemporary youth development researchers recognize the importance of an ecological perspective in understanding the dynamic relations between youth and their situational contexts in the process of development (Holt et al., 2020). Brofenbrenner's Ecological Systems Theory suggests the existence of four nested ecological levels that influence youth development: Microsystem (i.e., most proximate environment, such as a team, school classroom, youth program, or home), Mesosystem (i.e., extended systems and the interactions across them, such as family or school), Exosystem (e.g., community organizations and structures), and finally the Macrosystem (i.e., broader society and culture) (Bronfenbrenner, 1979). Based on Bronfenbrenner's conceptualization of development, YDPs can better support outcomes when they are intentionally implemented to promote ecological development and interactions within and across contexts (Duerden and Witt, 2010). At the microsystem level, Duerden and Witt suggest that programs be intentionally developed for the unique characteristics of the target population and desired outcomes, be culturally sensitive to make youth more comfortable, and provide meaningful opportunities to participate (Duerden and Witt, 2010). They further suggest that parental involvement in YDPs (Mesosystem) and organizational resources, staff training, and community partnerships (Exosystem) can both promote positive youth development within the context of YDPs and provide greater access to external developmental opportunities (Duerden and Witt, 2010). Finally, while researchers have developed an understanding of the associations among proximate ecological levels on individual youth development, what is less understood are the relationships among higher order ecological levels, which are imperative to program efficacy and sustainability, or the role of the macrosystem in influencing YDPs, especially within the context of sport (Edwards et al., 2013; Reverdito et al., 2017; Whitley et al., 2018). Bronfenbrenner's theory suggests that an integration of social and cultural context into the development of programs and organizations is critical to encouraging participation of youth in activities as well as providing a bridge to other systems (Hedegaard, 2009). The embeddedness of culture in programs may be particularly salient for the engagement of Latinx youth (Behnke et al., 2011).

Increasingly, researchers have emphasized a need to understand cultural and social contexts at the macrosystem level. Factors related to religion and culture, as well as socio-economic barriers, often influence the engagement of underrepresented youth in programs and whether PYD outcomes are achieved (Holt et al., 2011; Araki et al., 2013). YDPs must incorporate practices that recognize these contextual factors that either serve as facilitators or barriers to youth engagement to ensure the relevance of YDP activities as well as the efficacy of programs to support desired outcomes related to social change. Furthermore, it is recommended that agents working with youth within these contexts have a deep personal connection to the local conditions that are connected to these macrosystems (Lindsey et al., 2017).

Intervention approaches to improve educational outcomes among Latinx youth need to recognize the importance of cultural and social contexts in which those youth live. Young people are highly influenced by their social and cultural environments and interventions that focus solely on individual characteristics (e.g., motivation) without consideration of social contexts that influence youth or providing them with tools to navigate social environments may not facilitate successful outcomes (Duerden and Witt, 2010). The interaction of youth with their social environments is particularly important in the development of Latinx youth's educational outcomes and is specifically critical in the middle school years (Woolley et al., 2009). Research suggests that developing supportive microsystems (e.g., family, school personnel, and peers) as well as facilitating positive interactions among these microsystems (e.g., parental engagement at school) is associated with higher academic achievement among Latinx youth (Woolley et al., 2009). Additionally, maintaining a socially and culturally relevant ethnic identity is a significant macrosystem contextual consideration for engaging Latinx youth in YDPs (Rodriguez and Morrobel, 2004). Intervention programs that are culturally sensitive to the population they serve may help alleviate some barriers Latinx youth and parents may face to school engagement (e.g., language barriers, cultural expectations around school involvement, financial limits) (Hayes et al., 2015). Additionally, parent involvement has a positive correlation with Latinx high school retention (Schargel and Smink, 2014).

### Sport Approaches to Youth Development

From an ecological systems perspective, sport approaches to youth development have most often been considered from the microsystem perspective to understand how the structure or climate of a sport program may associated with developmental outcomes. Participating in sport programs can provide young people with positive physical, emotional, intellectual, and behavioral outcomes. Physical outcomes include cardiovascular fitness, improved muscular strength, endurance, flexibility, and bone structure (McKay et al., 2019). Sport participation may also promote the development of physical literacy or the skills needed to engage in physical activity across the lifespan (Edwards et al., 2017). Behavioral benefits can be seen in prosocial behavior (e.g., reduced chances of smoking, substance abuse, and increased physical activity), personal (e.g., owning responsibility for their actions) and social responsibility (e.g., community service) (Anderson-Butcher, 2019). Sport can also offer youth opportunities to experience challenges, enjoyment, increased self-efficacy, choice of autonomy (decision making), and decreased stress (Carreres Ponsoda et al., 2012). Youth sport participation has been associated with adult career achievement (Barker et al., 2014) and reduced school dropout and delinquent behavior (Chang et al., 2021). These findings highlight the potential for sport to enhance positive development, especially among underserved children.

Based on these positive outcomes associated with sport participation, sport provides a context that has been often associated with positive youth development (Lee and Martinek, 2013). It is often assumed that including sport in any program will result in positive outcomes for youth (Jones et al., 2017). However, sport participation does not inherently lead to positive youth development outcomes. Sports with poor structure and poor implementation can result in negative experiences for participants (Holt et al., 2020). Sport's ability to facilitate positive youth development only occurs when sport activities are intentionally designed and managed to achieve development goals (Kidd, 2008). The importance of sport's role in youth development is also limited without the inclusion of non-sport activities that may be more intentionally designed for PYD outcomes (Hartmann and Kwauk, 2011).

The context in which sport programs exist is also an important factor (Smith et al., 2019). Sport's efficacy to engage youth and deliver positive outcomes is dependent on being designed for specific social and cultural conditions and through engagement with local agents that are embedded in important macro-ecological systems in which youth engage (Spaaij, 2009; Cohen and Ballouli, 2018). While youth sport has always been socially constructed based on societal values, organized youth sport has become increasingly aligned with neoliberal ideologies that value privatization of programs (as opposed to public community programs), and promotes individual talent development, obedience to management, and competitive advantage in getting to the next level as youth sport's primary goals (Wilson and Hayhurst, 2009; Miracle and Rees, 2010). General organized youth sport approaches (e.g., competitive school sport, club sport, travel teams) that focus on competition, sport achievement, or sport development (as opposed to *for* development) may prioritize specialization and competitiveness at the expense of intentional overall developmental outcomes (Edwards, 2015). Additionally, organized youth sport has become increasingly exclusionary, requiring substantial financial resources, access to transportation, and parental time in order for youth to even participate and youth with lower socioeconomic backgrounds and from racial/ethnic minority groups often face considerable constraints to participation (Kuhn et al., 2021). Latinx youth in particularly often describe situations where local sport organizations and facilities implement targeted policies that prevent their access to programs and spaces to play soccer (Edwards et al., 2014; Lin et al., 2016).

Even when youth sport is intended as an intervention for specific developmental outcomes, they are often informed by deficit-based assumptions about youth (especially youth from racial and ethnic minority groups). These assumptions often guide programs in a way that focuses on improving individual attributes and avoiding risk behaviors. These programs often tend to be managed by agents from outside the community with less understanding of the social and cultural contexts relevant to youth (Rodriguez and Morrobel, 2004; Wilson and Hayhurst, 2009). Nevertheless, YDPs can effectively implement sport programming in multiple ways. Sport can be used as a leverage tool to support community health initiatives, as a hook to attract youth, or as a vehicle for program campaigns (Suárez-Orozco et al., 2010). Many organizations and initiatives have incorporated sports into their programming in efforts to strengthen education, to improve public health and community safety, and to develop social cohesion (Schulenkorf, 2012).

Within a sport context, participants' proximal relationships (e.g., with mentors, coaches, familial and non-familial adults, schools, and organizations) serve as important external assets that help facilitate or impede development (Bloom et al., 2005). In order to fully understand youth development, it is essential to examine the broader ecological contexts in which youth are developing and the effects that they experience (Carreres Ponsoda et al., 2012). Culturally-relevant community programs have been cited as an important intervention to keep Latinx youth in the educational system (Moncloa et al., 2021). A number of studies show that community organizations provide the context that allows Latinx students to connect with caring adults who support their educational aspirations, engagement, and attainment (Woolley et al., 2009). As a result of connections with adults, community-based programs assist with nurturing positive racial and ethnic identity development for students of color (Tsoi-A-Fatt and Harris, 2009).

## Methods

### Study Setting

Juntos (Spanish for “together”) was founded in 2007 at North Carolina State University. Juntos delivers programs in partnership with 4-H to 8th−12th grade Latinx students and their parents that focus on providing knowledge and resources for educational attainment. Founded in 1902, 4-H is the youth development program coordinated by the national Cooperative Extension System and the US Department of Agriculture (Home Page, 2022). Juntos developed four core areas: (1) **Monthly One-on-One Success Coaching and/or Mentoring** by an adult who monitors academics and coaches youth to achieve academic goals; (2) **Juntos 4-H Clubs** with a focus on tutoring, public speaking, life skills, healthy living, and community service; (3) **Juntos Family Engagement**, which includes a 6-week workshop series and other family events; and (4) **Juntos Summer Programming** that includes the Juntos Summer Academy and other local 4-H summer programs and events.

As part of program activities, some Juntos initiatives have incorporated sport activities. Sampson County's (NC) Juntos Program organizes a multi-county indoor soccer tournament, **Kicking it with Juntos (KIWJ)**. The tournament was open to middle and high school students from 6th to 9th grade who were enrolled in Sampson County schools and paid an $80 team registration fee. KIWJ was open to youth regardless of their affiliation with the overall Juntos program. The goal of KIWJ is to bring youth and family together for friendly competition and fundraise money to be able to support Juntos members to attend summer camps and educational events throughout the year. The tournament was also conceived as a recruitment mechanism for new Juntos members. Juntos members volunteer throughout the planning stages of the tournament (e.g., registration, marketing) and the day of the event (e.g., concessions, ticketing, crowd congestion, score keeping). Parents also engage in the tournament by volunteering as security and supervising main gate entrances.

The state-level Juntos program also hosts an annual soccer tournament called **Copa Unidos (CU)**, which is open to all North Carolina counties that have a functioning Juntos Program. CU places college students along with Latinx high school and middle school students on teams together to compete in the tournament. To be eligible to participate in the tournament, participants must be Juntos members. CU's ultimate goal is to utilize soccer to motivate middle and high school students to consider college. The interactions Juntos members have with college students, campus tours, and listening to motivational speakers are intended to get youth inspired and excited about college. The tournament is hosted on NC State's fields and in various facilities around campus. Juntos members are also paired with college student volunteers who act as mentors to the members during the event.

All CU participants paid a $5 registration fee that included lunch, drinks, and snacks. The Juntos college student planning committee administers the event and it receives some logistic and facility support from the university. College student volunteers assisted in officiating, checking in, lunch, non-player activities, media, and videography throughout the event day. Participant information for both tournaments are presented in Table 1.

**Table 1:** Participant demographics of Juntos tournaments.

	**Mean age (SD)**	**Number of participants**	
		**Male**	**Female**
Kicking it with Juntos	15.19 (2.00)	120	5
Copa Unidos	15.58 (1.56)	74	18
All Tournaments	15.36 (1.84)	194	23

### Research Design

Study protocols and data materials were approved by the NC State Institutional Review Board. This study consisted of semi-structured individual interviews, focus group interviews, and participant observations. This approach of multiple qualitative methods contributed to a more in-depth understanding of the research questions (Paul, 2005). All research volunteers who assisted in data collection received equal training prior to the start of the tournaments.

### Participant Recruitment

Participants for this study were recruited through both soccer tournaments. Two Latina graduate students (including the first author) recruited participants and collected the primary data. The two researchers started at opposite points of the field and each intercepted every 10^th^ player they encountered throughout the tournament. These research assistants interviewed players during lunch or downtime. In addition, 3 months after the tournaments, all Juntos participants and staff members were invited to participate in individual interviews or focus groups.

### Data Collection Procedures

#### Participant Observations

The lead author became an active member of the organizing committee in the early planning stages for both tournaments, becoming embedded in the program months in advance of the tournaments. The goal was to become more accepted as a natural part of the culture to assure that observations were more natural and authentic. This allowed the researcher to understand what was occurring in the early stages of the tournament planning, have a better grasp the program's implementation, and understand the decision-making process of staff and volunteers. Field notes were collected during regularly scheduled meetings and during the tournaments in order to document contextual information and aid in the preliminary coding of data (Phillippi and Lauderdale, 2018).

Two types of interviews were conducted to generate data for analysis. All interviews and focus groups at CU and for the focus group were conducted in English. During short, semi-structured interviews at KIWJ, five interviews were conducted in Spanish at the participant's request. The primary author, who is bilingual, translated the transcribed interviews into English for analysis. Short semi-structured interviews were conducted with ten tournament participants (7 males, 3 females, ages 12–19), and in-depth interviews were conducted with 6 county coordinators (all adult females) and 5 planning committee members (4 males, 1 female, ages 20–22). Tournament players participated in 5- to 10-min interviews the day of the tournament; county coordinators and planning committee members engaged in 30-min to 1-h interviews 2 months after the tournaments. A total of 26 interviews were transcribed and included in the analysis.

#### Short, Semi-structured Interviews

Throughout the course of the tournaments, researchers administered semi-structured interviews. Researchers along with the videography team interviewed participants. The videography team assisted in capturing the interview on video and taking photographs throughout the tournaments. Both interviewers underwent the same training in order to facilitate and deliver semi-structured interviews in the same way. Semi-structured interviews were administered as guided conversations; both researchers had a questionnaire protocol to use as guidance to help direct the conversation. Example items from the short, interview protocol included:

- Do you think having this soccer tournament has an impact for students, explain?
- What is your highlight of the tournament so far? How can this tournament be improved?
- Is this program a main component of Juntos?
- Do you believe this tournament aligns with Juntos mission/aims?

#### In-depth Interviews

In-depth interviews were administered after the event with staff members and adult volunteers. In-depth interviews took place during the fall of 2017. Each session was audio recorded and lasted 30-min to 1-h. The interviews were administered in person and a research volunteer assisted with taking notes and memos throughout the interviews. Questions were derived from a combination of studies (Corona et al., 2006; Coalter, 2007; Behnke and Aguilar, 2012) that have followed the trustworthy guidelines for qualitative research and support the study's research question. Example items from the interview protocols include:

- How does the soccer tournament help Juntos serve its target population?
- How does Juntos connect soccer to other activities within the program?
- In what ways is soccer important in helping Juntos achieve program outcomes?
- What do you intend to accomplish with sport included in the Juntos program?

In order to enable additional data generation, and based on the experiences of shorter answer responses in individual interviews with youth participants, one focus group was conducted with student volunteers who contributed to the planning and implementation of the soccer tournaments. A research volunteer assisted by taking notes throughout the focus group interview. The group interview followed the same interview guide as the individual interviews.

### Data Analysis

Audio recordings of the interviews were transcribed verbatim. The verbatim transcriptions were then sent back to participants to assure accuracy and relevance of what they shared.

Researchers received training on how to analyze given data and code for triangulation. According to Umaña-Taylor and Bámaca (Umaña-Taylor and Bámaca, 2004), when studying Latino populations multiple perspectives and interpretations of data should be considered. The data were triangulated by two research assistants who were familiar with qualitative data analysis and were both bilingual in English and Spanish. This was to (a) reconcile differences and theoretical perspectives, and (b) further analyze outcomes in order to validate the findings (Ramprogus, 2005). A confidential Google drive folder was shared with field experts, research assistants, and Juntos national and state directors to serve as an audit trail. The drive included detailed instructions on how to code trigger words or phrases and examples were listed to provide guidance. Coding followed an inductive, thematic process. During the open coding process, the data were categorized at the phrase or level, organized, and sorted using closed coding, followed by axial coding which placed these categories into similar topics (Charmaz, 2006). Additional codes and concepts emerged through the coding process and were discussed. In some cases, these additional codes were combined with existing codes. In other cases, new codes were added to the codebook. Three rounds of coding occurred followed by discussions amongst the coders. After three rounds, the researchers agreed that the threshold of empirical saturation had been reached (Lamont and White, 2005), with meaning emerging from respondents that presented a consistent pattern of responses to the research questions. Conceptual ordering was used to organize these initial themes into broader concepts based on similar dimensions (Strauss and Corbin, 1994). The first author met bi-weekly with research assistants over the course of 2 months to discuss the data as a way to enhance trustworthiness. These meetings allowed discussions about the process, codes, and emerging themes. Once data were evaluated and coded, results were compiled and disseminated to the Juntos Leadership Team and stakeholders. Pseudonyms were used to protect the identity of all research participants (i.e., player participants, county coordinators, volunteers, and planning committee members). These steps ensured the trustworthiness of the data (e.g., credibility, dependability, transferability, and confirmability).

### Researcher Positionality

In being transparent about the first author's possible research bias in this study, she identified herself as a Latinx female and as a sports fanatic. She previously played collegiate soccer throughout her undergraduate years and believed that sports served as a beneficial resource for youth development, particularly for minority, under-resourced youth. Personally, YDPs and SFD models influenced her own development growing up in an under-resourced, low SES environment. Her research bias also allowed her to be accepted within the community that she was working with. The way she reduced potential research bias was by adhering to established techniques for data collection and analysis, such as triangulating data by cross-checking outcomes with participants and research assistants and discussing the data with other members of the research team (Biddle et al., 2001).

## Results

Results are organized around themes that emerged in relation to the ecological systems associated with the Juntos' soccer programs and examined in light of associated best practices of YDPs. At the Microsystem level, sport was implemented to engage youth and connect to non-sport program outcomes, but divergent perceptions of goals among stakeholders potentially inhibited intentional implementation. At the Mesosystem level, sport provided a mechanism to engage in collaborative relationships and encouraged parental participation. At the Macrosystem level, sport celebrated Latino culture and attempted to address social barriers facing Latinx youth, but some aspects of culture may have created barriers to access for girls.

### Microsystem—Sport Implemented to Engage Youth and Connect to Non-sport Programs, but Divergent Perceptions of Goals Among Stakeholders Inhibited Intentional Implementation

Duerden and Witt suggest that at the microsystem level, YDPs should be intentionally designed for their target population, be inclusive and engaging, and provide meaningful opportunities to participate (Duerden and Witt, 2010). The overall efficacy of Juntos to achieve multiple program outcomes has been documented in the literature (Behnke and Aguilar, 2012; Behnke et al., 2019). The sport programs included embedded activities focused on higher education attainment in a comfortable environment. Specifically, the sport programs were designed to provide educational information and networking opportunities for youth in relation to higher education. Sport provided a more engaging and accessible space for this process. Nicole, a 20 year-old work-study college student who co-led CU, explained how educational opportunities were presented through networking and keynote speakers. CU participants were given a comfortable platform to develop mentoring relationships with college students they met during the tournament. CU served as an access point for player participants.

For example, Sophia, one of the participants, shared “…*you meet people that have the same passion education-wise, that you just feel connected. I've met college students that tell me you should go to this school because they have this program and stuff like that*.”

The program also ensured that programs were culturally relevant for their Latinx participants. Essentially the purpose was to encourage students and make them feel like college was obtainable by seeing and hearing from college students who have had similar life experiences. College student volunteers expressed how eager Juntos members were to talk to them and how the high school students were interested in learning about college. Nicolas, another 20 year-old, college student) described: “*they (Juntos youth) will ask you how long you have been in college, how and why I got there, how I pay for college, and it really opens their eyes when we have these types of conversations*.” In support, Jalisa, one of the county coordinators, added that a highlight of the CU tournament was seeing the students come back from the tournament and actually having an interest in higher education. There seemed to be an attitudinal change in the students. Jalisa expressed “… *for them it's like an eye opener, it's not just a dream but it can be done*.”

Because of the cultural importance of soccer in the Latino population, sport also functioned as a critical hook to some youth who otherwise may not have engaged in Juntos' non-sport programming. Erica (county coordinator for 4 years) shared that one of her students, Luis (17 year-old, junior in high school), came to the United States roughly 3 years ago and had never visited a college campus and had never really shown much interest in college or graduating high school. However, after hearing about CU, Luis wanted to attend and participate, due to his passion for playing soccer. Erica shared that after participating in the tournament she noticed an attitudinal and motivational shift in Luis's behavior and interest in education. County coordinators collectively noted that soccer tournaments significantly helped with recruitment and retention of participants. County coordinators were transparent about their direct recruitment use with both tournaments. Maria (county coordinator for 4 years) explained, “*I know that that's something (soccer tournaments) that I have used to promote the Juntos Program, I know kids get really excited and they look forward to it all year*.” Players also recruited one another. They informed their peers about all of the benefits of the tournament, and how much fun they would have. Marvin (15 year-old, junior in high school) explained, “*I went to the leadership program and I saw the video of Copa, and when I saw it I was like I have to come. Everyone seemed so together and then my peers kept telling me how amazing it was and that they met new people and everything, it just made me want to come even more*.”

While Juntos' sport programs provided many of the associated best program-level characteristicsfor PYD identified at the microsystem level, the divergent perceptions of objectives by relevant stakeholders may inhibit the intentional implementation of the programs. County coordinators, player participants, and planning committee members all had a, respectively, different understanding of the role of sport due to their individual contexts. County coordinators and other program staff were unclear about the intended purpose of sport in the sport programs and their role within the broader goals of Juntos. When asked about their opinion on the role of sport, some coordinators were at a loss for words, others tried connecting the role of sport to a 4-H physical activity initiative, and others simply stated that the leadership team did not communicate a clear purpose of including sport activities. Brenda, a county coordinator for 3 years, shared “*I have not been told of what the role of sport was going to be or given any direction as to prepare the team for anything*.” However, county coordinators universally suggested CU leaned more toward educational aspirations and networking, whereas KIWJ was a more competitive event focused on peer support for tournament implementation and outcomes.

Because the leadership did not articulate a clear intentional role for the sport programs, it may leave the role of sport more open to interpretation by staff and volunteers charged with implementing the programs, thus creating inconsistency and potentially mission drift. For example, the lack of clarity described above may create situations where the leadership of these programs draw upon more neoliberal values associated with youth sport, rather than sport aligned with PYD approaches. For instance, Jane (county coordinator for 5 years) suggested KIWJ was driven by competition as opposed to any positive developmental youth development outcomes. Interestingly, Jane's team in the tournament was eliminated in the semi-finals based on what she perceived as unfair substitution rules. She articulated that she and the youth on her team were upset by the officials' decisions. Either the approach of the tournament officials or Jane's expectations for competition may have potentially affected her perspective of the tournament's goals. Similarly, planning committee members from KIWJ believed that the goal of the tournament was to have everyone together to compete against one another and, in return, reinforce that everyone is a winner. However, some misconceptions were perceived related to whether competition or cooperation was the emphasis of KIWJ. This discrepancy among all participants at this tournament suggested a potential gap in communication among stakeholders that could affect participants' (and coordinators') motivations to support the non-sport content of the programs or be dismissive of the role of sport in supporting program goals, perceiving the sport component as a distraction to primary program goals.

### Mesosystem—Sport Provided a Mechanism to Engage in Collaborative Relationships and Encouraged Parental Participation

Critical to the process of development is relationships at the mesosystem level, and particularly building relationships with peers and the involvement of parents in programs to better understand their children's experiences (Duerden and Witt, 2010). Jane (county coordinator for 5 years) attended both tournaments and explained “*both tournaments generated different outcomes on my kids (Juntos members), but one thing that was constant was how satisfied they were about their parents watching them play, and the new friendships they made*.” Parent involvement was high within the Juntos sports programs, and in some cases, sport directly provided a catalyst for that involvement. Sara (a county coordinator for 2 years) had a student (Jose, 14 year-old sophomore in high school) express how impactful it was having his parents present at CU. Sara added that Jose had never played soccer, but his family always came together as a social event to watch soccer games like the World Cup. Jose tried out for his school's soccer team and did not make it, given the opportunity to participate in CU, Jose was the first to sign up.

“*Jose told me how proud and happy he was because his parents had never been able to see him play a game. I think having his parents present impacted him positively*.”—Sara, county coordinator.

Sampson County had more visible parent involvement compared to CU. Sampson County has limited staff members, which required a higher number of volunteers to deliver KIWJ. Parents' level of engagement leaned toward volunteering (maintenance, entrance security, concession stands) and watching the 2-day tournament. Parents and families filled the gymnasium bleachers to watch the middle school co-ed tournament Friday afternoon. For some parents, CU was the first time they saw their son or daughter play soccer. Brenda (county coordinator for 3 years) shared, “*I have a lot of parents that come out to support their children regardless of the drive if they do not have to work because they love the sport of soccer and want to support their child. This made parents feel really good and proud.”*

Both tournaments implemented a sense of comradery among youth. By opening the tournament to college students (CU) or other middle and high school students across Sampson County (KIWJ), Juntos members were able to build a network of support not only from their county but also with other peers across North Carolina. Mia, an eighth grader, shared how she was so excited to play at both tournaments because she would have the opportunity to meet new people at the CU tournament and see friends she made in previous Juntos events at KIWJ. Mia added: “*these soccer tournaments bring us together as a family, to play as a team and to get to know and trust each other.”* Tournament participants were able to develop their relationships among one another through these events.

### Macrosystem—Sport Celebrated Culture While Addressing Some Social Barriers for Youth

In order to engage youth in effective development processes, YDPs, particularly those working with Latinx youth, must be culturally informed (Behnke et al., 2011). County coordinators collectively suggested that both sport programs provided a context to build cultural connections for Latinx youth. Community is often highly valued within Latino culture. In many Latino families, *familismo* is a concept describing the process that fosters strong relationships among community and family members and placing family needs above individual needs (Delgado, 1995). The sense of community within the context of Latino culture was evident across the soccer tournaments. Across the two tournaments, player participants suggested tournaments encouraged unity, supported families, and assured cultural needs were met (e.g., bilingual staff, music, concession food and drinks). In support, Maria (county coordinator for 4 years) described how she witnessed her team shift from being classmates to being each other's support systems on and off the field.

“*I saw them not just being classmates, but they turned out to be friends and like when they had a goal scored against them, instead of the students saying it's your fault, they would hug the keeper or whomever and say we got this don't worry*.”—Maria, county coordinator.

Additionally, the context of soccer is one of high cultural importance in Latino communities, often providing a hook to engage youth, but also as a catalyst for community involvement. In addition, Brenda (county coordinator for 3 years) expressed that the high participation rates and attendance at soccer tournaments like Kicking it with Juntos sent a message to the Juntos youth, highlighting the support from their county. She added, “*attending Kicking it with Juntos tells the kids we are unified, that we are together and that we support them.”* This level of engagement across the community may have been less likely with a less culturally relevant activity than soccer. Brenda suggested that her Juntos youth are always committed to playing soccer as there is no greater cause they support than to come to play soccer and support their fellow Juntos members.

Although both tournaments were perceived to be culturally supportive, they each did so in a different manner based upon their resources or strategy for implementation. For instance, KIWJ seemed to have more flexibility to provide different cultural food at the concession stand during the tournament. This was mainly because parents volunteered to cook different cultural dishes for the Juntos club in Sampson County (Observation notes). Conversely, CU provided lunch for everyone present (the 91 players, college players, parents, volunteers, and staff members); thus, the combination of pizza and fruit was a favorable option to feed everyone as it was affordable and simple to order, and have it delivered during lunchtime (Observation notes). However, CU hosted their tournament in an open setting with access to tables and a sound system from the university. The sound system was used to play Latin music where player participants, volunteers, and parents were able to dance during lunch and in between any downtime. Through this, both programs were able to encourage a sense of unity, familial support, and attempted to meet cultural needs using different tactics.

Additionally, at the macrosystem level, YDPs must focus on addressing societal barriers and advocate for youth (Duerden and Witt, 2010). Sara (county coordinator for 2 years) suggested that these soccer tournaments enabled students to gain access and resources of the Juntos Program to which they would otherwise not have access. Sara has a student who came to the United States roughly 3 years ago. Sara highlights how sport in this case was used as a mechanism for the student to gain access to resources. Christian (19 year-old, junior in high school) came from El Salvador not familiar with anyone at school or in his community; however, Sara notes that he made the majority of his friends through soccer.

Sara added, “*Christian is one of these kids who never signed up to do anything extra. Him and his mom come and do the necessary but when I announce Copa I had not even gotten home and I had received an email from him saying that he wanted to sign up and every 5 days or so he would ask me do I need to do anything else, so it was definitely a way to link him in, draw him in through soccer*.”

Critical in navigating societal barriers to social mobility is development of networking and advocacy skills. It is common for Latinx youth to develop strong relationships among their inner circle; however, they lack strength on developing relationships outside this group, and to use these networks for advocacy efforts, often due to a lack of other forms of capital. It was suggested that CU challenged youth to network and develop relationships that can assist them in strengthening their relationship skills to when they need to speak with a University representative, potential employers, community leaders, and other stakeholders. Students developed mentoring relationships through team activities and were encourage to practice of getting outside their comfort zone. The soccer programs provided safe opportunities for youth to be exposed to and ask questions about higher-level systems (e.g., education system) and systemic barriers to social mobility. Erica (county coordinator for 4 years) added that the majority of her Juntos members returned with more confidence to speak up, engage, and use their voice to advocate for their community.

It should be noted that while Juntos' sport programs were open to all genders, girls were significantly underrepresented in participation numbers. This result may be a result of the soccer programs reflecting Latino cultural norms and societal barriers to Latinx girls' soccer participation generally (Wood, 2018; McGovern, 2021b). That said, girls who participated in the soccer programs reported that the team environment of the tournaments allowed them to feel less constrained by some of the cultural and social rules, especially for how girls should look and act, that might reflect some forms of expression outside of the sport context. As Sophia (16 year-old, junior in high school) pointed out, “*you're able to express your feelings in different types of ways not just what they think but they also get to know you a little bit better, by the way you talk with them and it's not just a relationship with judgments of how you look.”*

## Discussion

Bronfenbrenner's (Bronfenbrenner, 2005) Ecological Systems Theory emphasizes bidirectional influences between an individual's actions and their surrounding environmental context. Our findings suggested that the role of the soccer programs for Juntos primarily served to engage Latinx youth at the mesosystem level. These programs highlighted the opportunity sport approaches offer YDPs to engage Latino parents and youth. Parental involvement has a positive impact on reducing the likelihood of Latinx students dropping out of high school (Suárez-Orozco et al., 2010). Both tournaments attempted to alleviate the barriers to involvement faced by Latino parents (Conchas, 2015). Both Juntos tournaments incorporated parental engagement in various forms (e.g., volunteering: food, security, and coaching, officiating, and as spectators). Hoover-Dempsey and Sandler's (2005) model for parent involvement states that a parent is more likely to become involved in their child's sport experience if they perceive themselves to have the requisite knowledge and aptitudes. Soccer, an activity likely embraced and understood by Latino parents, provided an important bridge to engage parents with youth within the Juntos program.

Bronfenbrenner (1979) suggests that programs should connect youth in socially and culturally relevant ways to their broader community. Previous research highlights that programs that are culturally and ethnically affirming allow students to become better connected with their culture and may facilitate stronger connections to program outcomes (Wong, 2010; Cohen and Ballouli, 2018). In this study, research participants (players, county coordinators, and planning committees) suggested that they had a positive perception about the role of sport, specifically player participants noted the strong emotional connection and community building that occurred throughout the tournament days. Player participants highlighted that both tournaments made them feel included and part of a community and participants identified tournaments as events that allowed them to develop new friendships. Familismo has been suggested as a protective factor that keeps Latinx youth from being influenced by negative peer influences, drug use, and other risky behaviors (Reardon, 2016). This study indicated that the Juntos Program in North Carolina incorporated sport approaches that met the need for familism and development of social and cultural connections. The Latino community relies heavily on familism, which can be conceptualized through an assets framework as “bonding social capital” (Mathie and Cunningham, 2003). Parental support, peer support, and Latino community support was well-received by research participants. Player participants suggested that this sense of unity and pride resulted from this familism. The role of sport in strengthening the networks between young people and adults may not only improve individual outcomes through sport (Blom et al., 2013), but strengthened social capital networks among stakeholders may be critical in the transference of individual program goals to a broader focus on social change (Bruening et al., 2015). Some of the biggest perceptions for youth in these programs were having their parents present, reconnecting with friends from previous events, and meeting new individuals, like college students. Delgado (1995) suggests that Latinos' views on family expand to their communities, their role in the community and their preference to belong to and work in those groups. Both tournaments provided many opportunities for youth and parents to become involved, be present, and take full advantage of the resources available (i.e., networking/developing relationships). Juntos programs attempted to engage youth and increase community unity among participants.

At the macrosystem level, the soccer programs provided a culturally relevant hook to engage youth and their families and the program setting attempted to embrace Latino culture. The cultural importance of soccer for Latinx youth cannot be underestimated (McGovern, 2021a). Soccer offers Latinx youth and parents a familiar context, one in which they are comfortable in engaging. Despite the cultural attraction to soccer, Latinx youth may participate in organized versions of the sport at much lower rates than other racial and ethnic groups because of structural barriers related to the traditional youth sport system (McGovern, 2021a). Additionally, because many Latinx youth have described deliberate tactics by community sport organizations specifically designed to prevent their access to soccer fields (Edwards et al., 2014). Therefore, removing language and financial barriers, and providing an accessible setting to play soccer, maximized participation in the Juntos programs. However, despite being open to boys and girls, there was a significant gender disparity in participation. This finding is unsurprising considering past research that suggests coeducational sport offerings are typically hegemonic masculine environments in adolescence, with girls either excluded or choosing not to participate (Edwards et al., 2011). This issue may be exacerbated by soccer's historical hyper-masculine ethos in some Latino cultures (Wood, 2018). Furthermore, Latina girls may face more constraints to sport participation than other groups due to cultural biases as well as increased domestic responsibilities (McGovern, 2021b). Therefore, as designed, the Juntos soccer programs may have the unintended consequence of reducing the engagement and support of girls in their programs due to macrosystem effects of embedded cultural and social values of sport.

At the microsystem level, research participants had positive perceptions of the program but highlighted that both sporting events had unclear purposes. In particular, county coordinators described the confusion within the tournaments' objectives, stating that there was no clear purpose communicated to them. It is understandable for stakeholders to have different interpretations of the role of sport within the Juntos Program. However, programs can better provide settings and experiences that enable PYD to occur if program organizers, staff, and volunteers are intentional in how they implement activities (Perkins and Noam, 2007). Thus, sport approaches must establish clear goals and communicate these with relevant stakeholders.

The results of this study highlight a potentially missed opportunity for Juntos to maximize the role of sport in their program. Although both CU and KIWJ incorporated sport, these approaches were not always intentionally integrated into the events. A number of studies have shown that community organizations provide the context that allows Latinx students to connect with caring adults who support their educational aspirations, engagement, and attainment (Liou et al., 2009). However, if not all adults are clear on the intended purpose of the events, there may be a limit to the achievement of program outcomes. Particularly in youth sport designed to promote psychological and social outcomes, it is important that stakeholders, especially adults, are on the same page about the goals of sport (Jones et al., 2018). To emphasize inclusion, cooperation, and socialization while deemphasizing competition and performance may diverge with the value some individuals and organizations place on sport (Edwards, 2015). Thus, conflict may be inherent in these programs if doubts about the sport program's intended purpose are present. Youth who participate in sport programs that are designed for youth development outcomes may be more motivated and committed to the program design when intentional goals are clearly defined (Duerden and Witt, 2010). Additionally, this collective understanding may also promote increased cooperation and positive social interactions and help to deemphasize rivalries, aggression, and poor sporting behaviors that may undermine social and psychological goals (Fraser-Thomas and Côt, 2009; Jones et al., 2018).

### Limitations

This study had several limitations. First, like any qualitative study, the study is contextual to the setting. Thus, the findings in this study may not be representative of other Latino populations or YDP settings. In addition, working with the Latino community also known as the “hidden” or “hiding” community (e.g., undocumented, or involved in illegal activities) presents unique challenges for researchers to get participation due to fear or unfamiliarity (Deren et al., 2005). Nevertheless, extended involvement with the program allowed researchers to build rapport. Participant observation was beneficial to developing trust and familiarity with research participants; yet, it may also increase social desirability bias among research participants. Another potential limitation may be the duration of the tournaments as they were 1-day events and interviews were conducted immediately following the tournament. Research participants, specifically tournament player participants, may have been limited in the time they were given to fully reflect on the event. Based on the 1-day nature of this event, the applicability of the SFD framework may be limited. This limitation may have strained researchers from capturing participants' in-depth perspectives on their perceived role of sport in the Juntos Program. Additionally, the scope of data generation was limited both in terms of available participants and the length of time in the field to conduct interviews. Thus, while researchers agreed that empirical saturation was reached in data generation and analysis, it is possible that ongoing data generation with this population over a more prolonged period of engagement could yield additional insights into the process of sport in the context of youth development as well as additional themes. This study also does not investigate the potential impact of acculturation. The different levels of acculturation may impact how player participants view the role of sport within the Juntos Program (Garcia-Reid et al., 2005). Because soccer has unique cultural meaning in Hispanic countries, less acculturated respondents may value the role of the sport in any context differently from more acculturated respondents. Attempts to use soccer within the sport-for-development context intentionally for goals other than sport performance may be perceived differently based on level acculturation. Despite the limitations listed, this study had primary strengths that should be noted.

## Conclusion

According to Falicov (2005), the social climate of structural exclusion impacts both Latino parents and their children, as their sense of self-esteem decreases and opportunities to participate in the society at large are placed even further out of reach. Latinx youth development and academic achievement are often hindered due to poor living conditions, such as living in low SES communities, fear of crime and violence (Santacrose et al., 2021). However, YDPs have the potential to alleviate some barriers Latinx youth and parents may face in navigating some constraints to social mobility created by structural exclusion provided they recognize and incorporate processes that address the multi-layer ecological contexts of youth. This study examined how ecological systems for PYD functioned at different levels within a Latino-based community sport YDP. The cultural construct of familism in the mesosystem was identified as a strength for engaging Latino families. The Juntos soccer programs utilized sport approaches to incorporate the sense of familism throughout both tournaments.

The findings from the interviews suggested that the Juntos program's emphasis focused on the role of the microsystem (the participant's immediate environment such as teammates, coaches) and support of mesosystem (how the microsystem interacted to impact participants). On the one hand, the mesosystem (i.e., engagement with parents and peers) is important for supporting PYD in Latinx youth. However, as the results suggested, there is potentially an overreliance on sport at the microsystem level to deliver programmatic outcomes. Prior research is clear on the limitations of sport in that regard (Jones et al., 2020), especially (as results suggested) when the goals are not clearly defined by the stakeholders. Therefore, sport programs like this need to explore mechanisms for increased integration at the exosystem and macrosystem levels in order to better facilitate both structure for and sustainability of PYD outcomes. For example, some of the county coordinators' comments regarding the program's goals as facilitating lasting social change would rely on informal and formal structures in the exosystem (e.g., family obligations, influence of neighborhoods, community partnerships, sport organizations) and macrosystem (e.g., education policy, media, political ideology, social welfare systems) that do not directly affect youth but influences their development by interacting across systems. Ecological systems theory posits that collaborative systems approaches are necessary to move beyond a focus at the microsystem and mesosystem level to empower communities to take ownership of issues and unite to take action for lasting social change. Potentially through a better integration across the broader levels of the ecological system can SFD programs be more effective in attaining sustainable PYD outcomes.

## Data Availability Statement

The raw data supporting the conclusions of this article will be made available by the authors, without undue reservation.

## Ethics Statement

The studies involving human participants were reviewed and approved by North Carolina State University Institutional Review Board for Research with Human Subjects. Written informed consent to participate in this study was provided by the participants' legal guardian/next of kin.

## Author Contributions

MR, ME, JB, AB, and JC conceived and planned the study and contributed to the interpretation of the results. MR and AB carried out protocol development and data collection. MR wrote up the initial study as a MS thesis and ME took the lead in writing the manuscript with contributions from JB, AB, and JC. All authors provided critical feedback and helped shape the research, analysis, and manuscript.

## Conflict of Interest

The authors declare that the research was conducted in the absence of any commercial or financial relationships that could be construed as a potential conflict of interest.

## Publisher's Note

All claims expressed in this article are solely those of the authors and do not necessarily represent those of their affiliated organizations, or those of the publisher, the editors and the reviewers. Any product that may be evaluated in this article, or claim that may be made by its manufacturer, is not guaranteed or endorsed by the publisher.
